# The Essential Toxin: Impact of Zinc on Human Health

**DOI:** 10.3390/ijerph7041342

**Published:** 2010-03-26

**Authors:** Laura M. Plum, Lothar Rink, Hajo Haase

**Affiliations:** Institute of Immunology, Medical Faculty, RWTH Aachen University, Pauwelstrasse 30, 52074 Aachen, Germany; E-Mails: lplum@ukaachen.de (L.M.P.); lrink@ukaachen.de (L.R.)

**Keywords:** toxicity, zinc, essential trace element

## Abstract

Compared to several other metal ions with similar chemical properties, zinc is relatively harmless. Only exposure to high doses has toxic effects, making acute zinc intoxication a rare event. In addition to acute intoxication, long-term, high-dose zinc supplementation interferes with the uptake of copper. Hence, many of its toxic effects are in fact due to copper deficiency. While systemic homeostasis and efficient regulatory mechanisms on the cellular level generally prevent the uptake of cytotoxic doses of exogenous zinc, endogenous zinc plays a significant role in cytotoxic events in single cells. Here, zinc influences apoptosis by acting on several molecular regulators of programmed cell death, including caspases and proteins from the Bcl and Bax families. One organ where zinc is prominently involved in cell death is the brain, and cytotoxicity in consequence of ischemia or trauma involves the accumulation of free zinc. Rather than being a toxic metal ion, zinc is an essential trace element. Whereas intoxication by excessive exposure is rare, zinc deficiency is widespread and has a detrimental impact on growth, neuronal development, and immunity, and in severe cases its consequences are lethal. Zinc deficiency caused by malnutrition and foods with low bioavailability, aging, certain diseases, or deregulated homeostasis is a far more common risk to human health than intoxication.

## 1. Introduction

In the periodic table of the elements, zinc can be found in group IIb, together with the two toxic metals cadmium and mercury. Nevertheless, zinc is considered to be relatively non-toxic to humans [1]. This is reflected by a comparison of the LD_50_ of the sulfate salts in rats. According to the Toxnet database of the U.S. National Library of Medicine, the oral LD_50_ for zinc is close to 3 g/kg body weight, more than 10-fold higher than cadmium and 50-fold higher than mercury [2]. An important factor seems to be zinc homeostasis, allowing the efficient handling of an excess of orally ingested zinc, because after intraperitoneal injection into mice, the LD_50_ for zinc was only approximately four-fold higher than for cadmium and mercury [3]. In contrast to the other two metals, for which no role in human physiology is known, zinc is an essential trace element not only for humans, but for all organisms. It is a component of more than 300 enzymes and an even greater number of other proteins, which emphasizes its indispensable role for human health. Optimal nucleic acid and protein metabolism, as well as cell growth, division, and function, require sufficient availability of zinc [4].

In this review, we will give a brief summary of zinc homeostasis, followed by a description of the effects of acute zinc intoxication and the consequences of long-term exposure to elevated amounts of zinc. Besides systemic intoxication, there exists evidence for a physiological involvement of endogenous zinc in toxicity on the cellular level, e.g., regulating apoptosis in many different cell types, and having a prominent role in neuronal death. In the end, we will also briefly discuss the detrimental effects of zinc deficiency, because, unless they are exposed to zinc in the workplace or by accident, healthy individuals are at far greater risk of suffering from the adverse effects associated with zinc deficiency than from those associated with intoxication.

## 2. Zinc Homeostasis

The human body contains 2–3 g zinc, and nearly 90% is found in muscle and bone [5]. Other organs containing estimable concentrations of zinc include prostate, liver, the gastrointestinal tract, kidney, skin, lung, brain, heart, and pancreas [6–8]. Oral uptake of zinc leads to absorption throughout the small intestine and distribution subsequently occurs via the serum, where it predominately exists bound to several proteins such as albumin, α-microglobulin, and transferrin [9].

**Figure 1.**
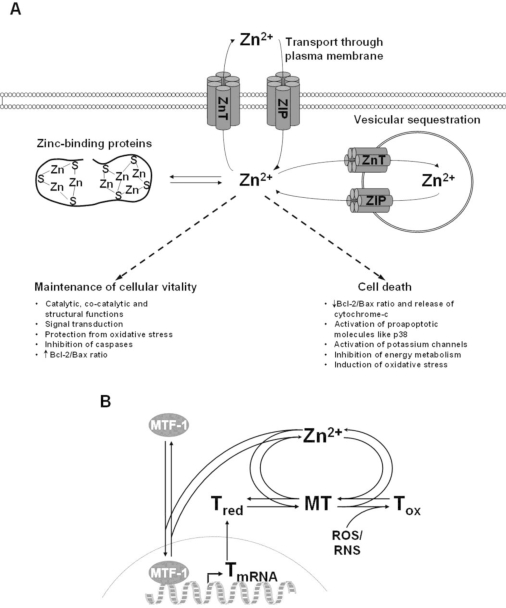
Cellular zinc homeostasis and its impact on cytotoxicity (A) Cellular zinc homeostasis is mediated by three main mechanisms. First, by transport through the plasma membrane by importers from the Zip-family, and export proteins from the ZnT-family. Second, by zinc-binding proteins such as metallothionein. Third, by transporter-mediated sequestration into intracellular organelles, including endoplasmic reticulum, Golgi, and lysosomes. Tight control of zinc homeostasis is required for maintenance of cellular viability, whereas deregulation leads to cell death. (B) A particular role in intracellular zinc homeostasis is played by the metallothionein/thionein-system. Free and loosely bound zinc ions are bound by the apo-protein thionein (Tred), to form metallothionein (MT). Elevated levels of free zinc ions can bind to zinc finger structures of the metal-regulatory transcription factor (MTF)-1, thus inducing the expression of thionein. Additionally, oxidation of thiols by reactive oxygen (ROS) or nitrogen (RNS) species triggers the formation of the oxidized protein thionin (Tox) with concomitant release of zinc.

On the cellular level, 30–40% of zinc is localized in the nucleus, 50% in the cytosol and the remaining part is associated with membranes [4]. Cellular zinc underlies an efficient homeostatic control that avoids accumulation of zinc in excess (see also Figure 1a). The cellular homeostasis of zinc is mediated by two protein families; the zinc-importer (Zip; Zrt-, Irt-like proteins) family, containing 14 proteins that transport zinc into the cytosol, and the zinc transporter (ZnT) family, comprising 10 proteins transporting zinc out of the cytosol [10].

The same transporter families also regulate the intracellular distribution of zinc into the endoplasmic reticulum, mitochondria, and Golgi. In addition, many mammalian cell types also contain membrane-bound vesicular structures, so-called zincosomes. These vesicles sequester high amounts of zinc and release it upon stimulation, e.g., with growth factors [11,12].

Finally, metallothioneins (MTs) play a significant role in zinc homeostasis by complexing up to 20% of intracellular zinc (Figure 1b) [13,14]. MTs are ubiquitous proteins, characterized by a low-molecular weight of 6–7 kDa, high cysteine content, and their ability to complex metal ions. One MT molecule can bind up to seven zinc ions. Through different affinities of the metal ion binding sites, it can act as a cellular zinc buffer over several orders of magnitude [15]. Dynamic regulation of cellular zinc by MT results from the synthesis of the apo-form thionein (T) in response to elevated intracellular zinc levels by triggering the metal response element-binding transcription factor (MTF)-1 [16]. In addition, oxidation of cysteine residues can alter the number of metal binding thiols, connecting redox and zinc metabolism. An in-depth discussion of this complex subject can be found in a recent review [17].

## 3. Exposure to Zinc

**Figure 2.**
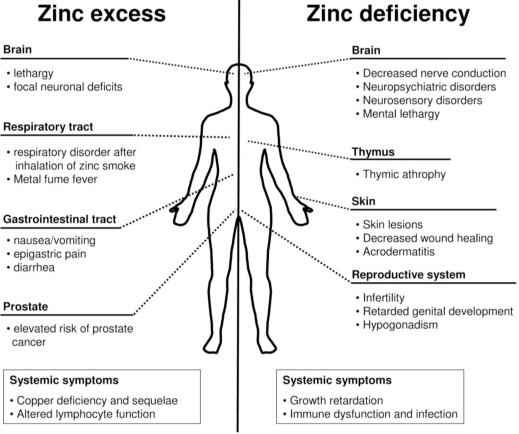
Comparison of the effects of zinc intoxication *versus* deficiency. Intoxication by excessive exposure to, or intake of, zinc (left hand side), and deprivation of zinc by malnutrition or medical conditions (right hand side), have detrimental effects on different organ systems. Effects that could not be attributed to a certain organ system or affect several organs are classified as systemic symptoms.

There are three major routes of entry for zinc into the human body; by inhalation, through the skin, or by ingestion [18]. Each exposure type affects specific parts of the body (Figure 2) and allows the uptake of different amounts of zinc.

### 3.1. Exposure by Inhalation

Inhalation of zinc-containing smoke generally originates from industrial processes like galvanization, primarily affecting manufacture workers. In addition, military smoke bombs contain zinc oxide or zinc chloride, making soldiers a group in which several cases of inhalation of zinc-containing fumes were described. For example, Homma and colleagues reported a case of two soldiers who developed adult respiratory distress syndrome (ARDS) upon exposure to a zinc chloride-containing smoke bomb [19]. The two men died 25 and 32 days after the accident, respectively. Another soldier was exposed to concentrated zinc chloride for several minutes during military training [20]. He also developed ARDS 48 h after exposure. After tracheal intubation and mechanical ventilation for eight days, he left the hospital, and four months after the incident he returned to work without any respiratory disorder [20]. There are a few additional reports of related incidents with smoke bombs having similar effects on the respiratory tract [21,22].

However, in none of the incidents there was unequivocal evidence that zinc was the main cause for the respiratory symptoms. Not only was no information about the concentrations available, but also the inhaled smoke contained several other ingredients besides zinc chloride. In addition, zinc chloride is generally caustic, so the effects could have risen from the specific properties of the compound, rather than being a direct effect of zinc intoxication.

The most widely known effect of inhaling zinc-containing smoke is the so-called metal fume fever (MFF), which is mainly caused by inhalation of zinc oxide. This acute syndrome is an industrial disease which mostly occurs by inhalation of fresh metal fumes with a particle size <1 μm in occupational situations such as zinc smelting or welding [23]. Symptoms of this reversible syndrome begin generally a few hours after acute exposure and include fever, muscle soreness, nausea, fatigue, and respiratory effects like chest pain, cough, and dyspnea [24]. The respiratory symptoms have been shown to be accompanied by an increase in bronchiolar leukocytes [23]. In general, MFF is not life-threatening and the respiratory effects disappear within one to four days [25].

Development of MFF is connected to the exposure level, but very little data is available concerning the zinc concentrations that trigger this syndrome [26]. Two volunteers developed MFF as a consequence of acute inhalation (10–12 minutes) of 600 mg zinc/m^3^ as zinc oxide [27]. Hammond and colleagues reported about workers who had shortness of breath and chest pain 2–12 hours following exposure to 320–580 mg zinc/m^3^ as zinc oxide [28]. Only small changes in forced expiratory flow were observed after exposure to 77 mg zinc/m^3^ (15–30 minutes) as zinc oxide [29]. Several reports of exposures to lower concentrations of zinc oxide (14 mg/m^3^ for eight hours, 8–12 mg zinc/m^3^ for up to three hours and 0.034 mg zinc/m^3^ for six to eight hours) did not result in symptoms of metal fume fever [28,30,31]. Today, the permissible exposure limit according to the Occupational Safety and Health Administration (OSHA) is 5 mg/m^3^ for zinc oxide (dusts and fumes) in workplace air during an 8-hour workday, 40-hour work week [32].

### 3.2. Dermal Exposure

Dermal absorption of zinc occurs, but the number of studies is limited and the mechanism is still not clearly defined. Agren and colleagues pointed out that the pH of the skin, the amount of zinc applied, and its chemical speciation influence the absorption of zinc [33,34].

In a study in which a 25% zinc oxide patch (2.9 mg/cm^2^) was placed on human skin for 48 hours, there was no evidence of dermal irritation [33]. In another study comparing the dermal effect of different zinc compounds in mice, rabbits, and guinea pigs, zinc chloride was clearly the strongest irritant, followed by zinc acetate, causing moderate, and zinc sulfate, causing low irritations. Consistent with the study by Agren, zinc oxide did not show any irritant effect on skin [35].

As mentioned above, zinc chloride is caustic, and the irritation does not necessarily indicate a toxic effect of zinc. In contrast to a potentially harmful effect of zinc on skin, it should be noted that zinc is a well-known supplement for topical treatment of wounds and several dermatological conditions [34,36–38]. Based on the existing data, it can be concluded that dermal exposure to zinc does not constitute a noteworthy toxicological risk.

### 3.3. Oral Exposure

Due to its nature as an essential trace element, oral uptake of small amounts of zinc is essential for survival. The recommended dietary allowance (RDA) for zinc is 11 mg/day for men and 8 mg/day for women [39]. Lower zinc intake is recommended for infants (2–3 mg/day) and children (5–9 mg/day) because of their lower average body weights [39]. This is significantly below the LD_50_ value, which has been estimated to be 27 g zinc/day humans based on comparison with equivalent studies in rats and mice [18]. In general, uptake of such an amount is unlikely, because approximately 225–400 mg zinc have been determined to be an emetic dose [40]. However, there is one published report of a woman who died after oral intake of 28 g zinc sulfate. After ingestion, she started vomiting and developed tachycardia as well as hyperglycemia. She died five days later of hemorrhagic pancreatitis and renal failure [41].

Immediate symptoms after uptake of toxic amounts of zinc include abdominal pain, nausea, and vomiting. Additional effects include lethargy, anemia, and dizziness [42]. Particular effects of excessive oral zinc exposure are discussed in detail below.

#### Gastrointestinal Effects

The gastrointestinal tract is directly affected by ingested zinc, before it is distributed through the body. Therefore, multiple gastrointestinal symptoms after oral uptake of zinc have been reported. Brown *et al.* described several cases in which high zinc ingestion resulted from storage of food or drink in galvanized containers. Ingestion was caused by the moderately acidic nature of the food or drink, enabling the removal of sufficient zinc from the galvanized coating. The resulting symptoms included nausea and vomiting, epigastric pain, abdominal cramps, and diarrhea [40].

In a study by Samman and Roberts, symptoms such as abdominal cramps, vomiting and nausea occurred in 26 of 47 healthy volunteers following ingestion of zinc sulfate tablets, containing 150 mg elemental zinc, for six weeks [43]. However, similar doses have been used in several other zinc supplementation studies without comparable side effects [44].

In addition to zinc sulfate, other zinc compounds like zinc oxide and zinc gluconate also have a similar impact on the gastrointestinal system [45–47]. A 39-year-old man showed nausea, vomiting, and abdominal pain six hours after ingesting 150 g of a 10% zinc oxide lotion, but without signs of systemic toxicity. Furthermore, he developed gastroduodenal corrosive injury. The symptoms persisted for three days and on the fifth day of admission, the corrosive injury showed regression without cicatrization [47].

#### Zinc-Induced Copper Deficiency

Taking up large doses of supplemental zinc over extended periods of time is frequently associated with copper deficiency [48–50]. This correlation seems to be caused by the competitive absorption relationship of zinc and copper within enterocytes, mediated by MT. The expression of MT is upregulated by high dietary zinc content, and MT binds copper with a higher affinity than zinc. Consequently, available copper ions are bound by MT and the resulting complex is subsequently excreted [51,52]. Oestreicher and Cousins stated that the dietary intake of different doses of copper and zinc did not significantly alter the absorption of the other metal, as long as they were given at the same ratio, irrespective if 1 mg/kg copper and 5 mg/kg zinc, or up to 36 mg/kg copper together with 180 mg/kg zinc were given [53]. Nevertheless, copper absorption is depressed when zinc is given in high excess over copper [54].

Frequent symptoms of copper deficiency include hypocupremia, impaired iron mobilization, anemia, leukopenia, neutropenia, decreased superoxide dismutase (SOD) (particularly erythrocyte SOD (ESOD)), ceruloplasmin as well as cytochrome-c oxidase, but increased plasma cholesterol and LDL:HDL cholesterol and abnormal cardiac function [55–57].

Furthermore, Irving and colleagues reported the case of a 19-year old woman who was supplemented with two doses of 50 mg zinc per day as part of a treatment of Hallervorden–Spatz syndrome, leading to a total daily intake of about 121.25 mg of zinc for more than 5 years, corresponding to approximately 15 times the RDA. Her daily intake of copper was 2 mg, which was approximately twice the RDA. As a result, she was markedly anemic and had severe neutropenia. Zinc-induced copper deficiency was confirmed by elevated serum zinc and low copper and ceruloplasmin serum levels. Four weeks after zinc therapy was stopped, all hematological and trace-metal parameters showed strong trends toward normalization and were normal after eight months [58].

Prasad and colleagues reported several cases of patients with sickle cell anemia who received 150 mg zinc/day and consequently showed low plasma copper, low ceruloplasmin, leukopenia, and anemia [59]. Another case report described a 31-year-old schizophrenic man who had been ingesting coins for 10 years [60]. He entered the hospital with symptoms including nausea, vomiting, and abdominal pain. Furthermore, profound anemia, neutropenia, and virtually absent serum copper and ceruloplasmin levels together with elevated zinc levels were diagnosed. Upon X-ray examination a large number of coins (totaling $22.50) were identified and surgically removed. Following the surgery, anemia and copper deficiency rapidly resolved. His copper deficiency was attributed to the ingestion of pennies, which since 1982 are composed of 98% zinc and 2% copper [60]. Several additional reports of zinc-induced copper deficiency leading to anemia and several other cytopenias were reviewed by Fiske and colleagues [55].

The mechanism by which copper deficiency induces anemia is based on the requirement of copper for several enzymes involved in iron transport and utilization and, therefore, in heme synthesis. For example, ceruloplasmin is a ferroxidase that binds copper and converts ferrous to ferric iron, allowing it to bind to transferrin and be transported. Cytochrome-c oxidase is also dependent on copper, and is required for the reduction of ferric iron to be incorporated into the heme molecule [61–63]. In addition to interference with heme synthesis, copper deficiency causes approximately 85% reduction of ESOD in the red blood cell (RBC) membrane, decreasing RBC survival time [64].

Whereas a recent meta-analysis found no general effect of zinc supplementation on serum lipoproteins [65], it may occur as a consequence of disturbed copper homeostasis. Copper deficiency is related to alterations of serum cholesterol levels [57]. In healthy men, a daily intake of 160 mg zinc/day decreased HDL cholesterol significantly [66,67]. Also, young women who ingested 100 mg zinc/day showed a reduction in HDL cholesterol [68]. A study with 24 men who were fed omnivorous diets that were deficient in copper (0.89 mg) and high in zinc (21.4 mg), *i.e.*, a Zn:Cu ratio of 23.5, showed low plasma copper, ESOD and HDL cholesterol, while LDL cholesterol was elevated [69]. This study was stopped after 11 weeks because four participants experienced cardiac abnormalities. Klevay and colleagues fed one man an omnivorous diet providing a Zn to copper ratio ≥ 16 for 105 days. Plasma copper and ceruloplasmin decreased, whereas total cholesterol and LDL cholesterol increased [70]. This experiment was ended when arrhythmia was detected. Taking into account several additional studies, Sandstead suggested that cardiac abnormalities were associated with Zn to copper ratios ≥16 [57].

#### Zinc Supplementation and Cancer

Whereas several other metals are well-known carcinogens, zinc is not generally considered to be a causative agent for cancer development. In contrast, displacement of zinc from zinc-binding structures, e.g., finger structures in DNA repair enzymes, may even be a major mechanism for carcinogenicity of other metals such as cadmium, cobalt, nickel, and arsenic [71].

One well investigated example in which an involvement of zinc in cancer development was suggested is prostate cancer. Notably, zinc levels in prostate adenocarcinoma are significantly lower than in the surrounding normal prostate tissues, suggesting an implication of zinc in the pathogenesis and progression of prostate malignancy [72–74]. This is based on a down regulation of the zinc transporter Zip1, which is responsible for zinc uptake and accumulation in prostate cells [75,76].

Men with moderate to higher zinc intake may have a lower risk for prostate cancer, but the opposite may be true at extremely high doses and long-term supplementation [77]. A study by Leitzmann and colleagues examined the association between supplemental zinc intake and prostate cancer risk among 46,974 U.S. men. During 14 years, 2901 new cases of prostate cancer were observed, of which 434 were diagnosed as advanced cancer. Supplemental zinc intake at doses of up to 100 mg/day did not cause a higher prostate cancer risk, whereas long-term supplementation with higher doses increased the relative risk 2.9-fold [78]. This increased risk may not be due to direct carcinogenicity of zinc, because it is known that immunosuppression significantly increases the incidence of cancer, and, as discussed in the following paragraph, high doses of zinc can be immunosuppressive.

#### Immunological Effects

Sufficient availability of zinc is of particular importance to the immune system. Thereby, it plays a key role in multisided cellular and molecular mechanisms [79,80]. For instance, zinc influences the lymphocyte response to mitogens and cytokines, serves as a co-factor for the thymic hormone thymulin, and is involved in leukocyte signal transduction [81–83]. An influence of zinc excess on T cell function was observed in several *in vitro* studies. In cell culture, very high zinc concentrations (above 100 μM) in a serum-free culture medium stimulate monocytes to secrete pro-inflammatory cytokines [84], but actually inhibit T cell functions. In general, T cells have a lower intracellular zinc concentration and are more susceptible to increasing zinc levels than monocytes [85,86]. Also, *in vitro* alloreactivity was inhibited in the mixed lymphocyte reaction (MLC) after treatment with more than 50 μM zinc [87]. A similar inhibition was observed when the MLC was done *ex vivo* with cells from individuals that had been supplemented with 80 mg zinc per day for one week, indicating that zinc supplementation has the potential to suppress the allogeneic immune response at relatively low doses [88].

An *in vivo* study supported the finding that zinc excess can affect lymphocyte function. 83 healthy volunteers ingested 330 mg zinc/ day in three doses for a month. The treatment had a small but significant influence on the lymphocyte response to the mitogens phytohemagglutinin (PHA) and Concanavalin A (Con A). Interestingly, it was observed that zinc had an immuno-regulatory influence, *i.e.*, it decreased the lymphocyte response in high responders and had an enhancing effect on low responders [89].

## 4. The Role of Zinc in Cell Death

In addition to the systemic toxic effects of zinc, this metal is also involved in the regulation of live and death decisions on the cellular level. First, we will discuss its role in apoptosis. Second, we will focus on an organ where zinc toxicity has been investigated in great detail, the brain.

### 4.1. Impact of Zinc on Apoptosis

The exact role of zinc in the regulation of apoptosis is ambiguous. A variety of studies indicate that, depending on its concentration, zinc can either be pro- or anti-apoptotic, and both, zinc deprivation and excess, can induce apoptosis in the same cell line [90–93].

The induction of apoptosis by high levels of intracellular zinc has been shown in different tissues and cell types [93–95]. Reports indicate that accumulation of intracellular zinc, either as a consequence of exogenous administration or release from intracellular stores by reactive oxygen species or nitrosation, activates pro-apoptotic molecules like p38 and potassium channels, leading to cell death [93,96–98]. Increased intracellular zinc levels may also induce cell death by inhibition of the energy metabolism [99,100].

Sensitive targets of zinc toxicity are the anti-apoptotic Bcl-2-like and pro-apoptotic Bax-like mitochondrial membrane proteins. In context of its apoptosis-inducing properties, zinc has been shown to increase the expression of Bax, leading to a decrease in the Bcl-2/Bax ratio [101]. As a consequence, dissipation of the mitochondrial membrane potential leads to the release of cytochrome-c from mitochondria into the cytosol [96,102–105].

The anti-apoptotic properties of zinc likely comprise two main mechanisms. First, zinc limits the extent of damage induced during oxidative stress, thereby suppressing signaling pathways resulting in apoptosis. Second, zinc directly affects several proteins and pathways that regulate apoptosis.

Consistent with the first issue, zinc deficiency has been shown to induce oxidative stress [106–108]. Mechanisms by which the redox-inert zinc protects cells against oxidative damage seem to include its property to protect sulfhydryl groups in proteins from oxidation [109]. Furthermore, by stabilizing lipids and proteins, zinc can preserve cellular membranes and macromolecules from oxidative damage. On the other hand, it has to be noted that elevated availability of zinc may also induce oxidative stress, and its impact on redox homeostasis may either be protective or promoting, depending on its availability [17].

With regard to the second mechanism, interaction of zinc with several apoptosis-regulating molecules has been reported. Zinc is a potent caspase-3 inhibitor [110] with an IC_50_ below 10 nM [111]. Furthermore, inhibition of caspases-6, -7, and -8 at low zinc concentrations was also shown, with caspase-6 being the most sensitive of the three [112].

Zinc deficiency can also induce apoptosis by disrupting growth factor signaling molecules such as ERK and Akt [113]. Other molecular targets for zinc are the anti-apoptotic Bcl-2-like and pro-apoptotic Bax-like mitochondrial membrane proteins. Zinc has been shown to increase the Bcl-2/Bax ratio, thereby increasing the resistance of the cells to apoptosis [114]. Consistent with this, in a study by Zalewski and colleagues apoptosis was induced in premonocytic cells by treatment with hydrogen peroxide. Supplementation with 1 mM zinc increased the ratio of Bcl-2 to Bax resulting in the inhibition of active caspase-3 and reduction of apoptosis [115]. Zinc-mediated apoptosis is abrogated by chelation with TPEN [116]. This is not undisputed, because it has also been shown in another study that zinc can increase the expression of Bax, leading to an decreased Bcl-2/Bax ratio and the release of cytochrome-c from mitochondria [101].

The influence of zinc on apoptosis is very complex and data are in part even contradictory. Amongst others, variables in this complex network are tissue and cell type, zinc concentration, expression of zinc transporters and zinc-binding proteins, other environmental circumstances like oxidative or nitrosative stress, and the involvement of multiple molecular targets with opposing functions.

### 4.2. Role of Zinc in Neuronal Death

A prominent and well investigated example for the control that zinc exerts on survival on the cellular level is the brain. This will now be discussed in more detail as an example of the mechanisms by which zinc can influence cellular survival.

Normally, homeostatic mechanisms should prevent zinc from accumulating in the brain to reach toxic concentrations as a result of excessive oral ingestion. However, there are reports of neurological symptoms following zinc intoxication, e.g., of a boy who showed lethargy and focal neurological deficits three days after he ingested 12 g of metallic zinc [117].

Many studies indicate that zinc acts as a neuromodulator [118–121]. On the other hand, experimental evidence indicates that endogenous zinc might be a relatively potent, rapidly acting neurotoxin, and, to a lesser extent, also a gliotoxin [122–126].

Zinc is stored in and released from vesicles in presynaptic terminals of a specific subset of neurons that also releases glutamate. Therefore, these neurons are defined as “gluzinergic” neurons [119, 127]. Zinc can be released from presynaptic terminals during synaptic transmission, enabling it to enter postsynaptic somata and dendrites of cells via zinc-permeable ion channels [105]. These channels include NMDA (N-methyl-D-aspartate)-gated channels [128], voltage-gated calcium channels [129,130] and the calcium-permeable AMPA (α-amino-3-hydroxy-5-methyl-4-isoxazole propionic acid)/kainate channel [131,132].

In addition to being sequestered in vesicles of presynaptic terminals in the gluzinergic neurons, zinc can also be bound to MT, especially MT-III, in perikarya as well as being taken up by mitochondria [133]. The MT-III isoform is found only in the brain and it is abundant in the gluzinergic neurons [134,135].

Exposure to 300–600 μM zinc for 15 minutes results in extensive neuronal death in cortical cell culture [136]. Considering that neurons store high amounts of free zinc in their terminals [137] that are released upon depolarization [138,139], zinc may play an active role in neuronal injury. Furthermore, membrane depolarization, which is associated with acute brain injury [140], greatly increases the potency of zinc to act as a neurotoxin [141]. Weiss *et al*. confirmed this by showing that depolarization with high concentrations (25 mM) of potassium media requires just a five minute-exposure to 100 μM zinc to kill all neurons in cortical cell culture [131].

Zinc has been described as a critical component of the excitotoxic cascade occurring after ischemia, seizures, and head trauma [141–143]. The first study providing evidence that zinc accumulation may play a role in the selective death of dentate hilar neurons after global ischemia in rats was done by Tonder and colleagues [144]. In the meantime, zinc accumulation in dying or dead neurons has not only been shown in the hippocampal hilar region, but also in all brain regions damaged in global ischemia such as hippocampal CA1, neocortex, thalamus, and striatum [145]. Consistent with the hypothesis that zinc-accumulation may lead to neuronal cell death, this event was prevented by the intraventricular injection of the zinc-chelating agent CaEDTA [145].

Zinc release and accumulation of zinc ions was also observed in a rat model of traumatic brain injury, where Suh and colleagues showed that trauma is associated with loss of zinc from presynaptic boutons and appearance of zinc in injured neurons. Again, neuroprotection occurred by intraventricular administration of a zinc chelator [146].

For some time, vesicular zinc was thought to be the only releasable pool of zinc in the brain [127]. This led to the assumption that the zinc ions accumulating in injured neurons must be entirely of presynaptic origin [127], but when ZnT-3 knock-out mice were investigated, which lack histochemically reactive zinc in synaptic vesicles, they still showed zinc accumulation in degenerating neurons, pointing toward sources other than synaptic vesicular zinc [147]. Alternative dynamic zinc sources might be MT-III as well as mitochondrial stores in the postsynaptic neurons [148,149].

Although zinc is redox-inactive in biological systems and exists only as a bivalent cation, there is evidence that zinc toxicity in neurons is mediated mainly by oxidative stress [141]. Zinc-induced cell death is associated with increased levels of reactive oxygen species in neurons [150,151]. In addition, free-radical-generating enzymes like NADPH oxidase are induced and activated by exposure to zinc [152]. Finally, zinc-induced cell death has been shown to be attenuated by various antioxidant interventions [96,153].

Besides oxidative stress, nitrosative stress can also affect zinc-induced neuronal injury. Nitric monoxide plays a crucial role in zinc toxicity by releasing zinc ions from MT [154], and inhibition of nitric oxide synthase significantly reduces zinc release from brain slices during oxygen and glucose deprivation [155]. Consistent with this, Frederickson and colleagues observed that nitric oxide also rapidly releases zinc from presynaptic terminals [156].

In addition to the impact of zinc on apoptosis discussed above, zinc-induced apoptosis in neurons might be based on two additional mechanisms. First, zinc-exposed neurons show an induction of the neutrophin receptor p75^NTR^ and p75^NTR^-associated death executor (NADE) [157], a combination that can trigger caspase activation and apoptosis [158]. Second, high intracellular zinc concentrations trigger dysfunction of neuronal mitochondria, resulting in the release of pro-apoptotic proteins such as cytochrome-c and apoptosis-inducing factor (AIF) [148].

Although the release of intracellular zinc triggers neuronal apoptosis [96,159,160], indicators of necrosis such as cell body swelling and destruction of intracellular organelles have also been observed [96,150], indicating that zinc-induced neuronal cell death might encompass both apoptotic and necrotic mechanisms [143]. Taken together, alterations of neuronal zinc homeostasis have a profound influence on cellular survival during acute insults, and zinc chelators are discussed as potential therapeutic agents for the treatment of stroke [161].

It seems likely that zinc is also involved in neurodegenerative diseases, e.g., zinc and a deregulated zinc homeostasis could be important to onset and progression of Alzheimer’s disease [162]. Here, the use of metal chelators such as clioquinol to restore normal neuronal zinc homeostasis has shown promising results *in vivo* [163].

## 5. Zinc Deficiency

As discussed above, systemic zinc toxicity is not a major health problem. On the other hand, due to its essentiality, a lack of this trace element leads to far more severe and widespread problems. Both, nutritional and inherited zinc deficiency generate similar symptoms [164], and clinical zinc deficiency causes a spectrum from mild and marginal effects up to symptoms of severe nature (Figure 2) [165].

Human zinc deficiency was first reported in 1961, when Iranian males were diagnosed with symptoms including growth retardation, hypogonadism, skin abnormalities, and mental lethargy, attributed to nutritional zinc deficiency [166]. Later studies with some Egyptian patients showed remarkably similar clinical features [167]. Additional studies in the ongoing years manifested zinc deficiency as a potentially widespread problem in developing as well as in industrialized nations [168].

Severe zinc deficiency can be either inherited or acquired. The most severe of the inherited forms is acrodermatitis enteropathica, a rare autosomal recessive metabolic disorder resulting from a mutation in the intestinal Zip4 transporter [169]. Symptoms of this condition include skin lesions, alopecia, diarrhea, neuropsychological disturbances, weight loss, reduced immune function, as well as hypogonadism in men, and can be lethal in the absence of treatment [170].

Acquired severe zinc deficiency has been observed in patients receiving total parental nutrition without supplementation of zinc, following excessive alcohol ingestion, severe malabsorption, and iatrogenic causes such as treatment with histidine or penicillamine [165]. The symptoms are mostly similar to those arising during acrodermatitis enteropathica.

Some reports indicate the existence of another group of inherited disorders of zinc metabolism. They lead to baseline zinc plasma levels above 300 μg/100 mL, more than three times the physiological level, while iron and copper levels stay normal [171–173]. Even though this exceeds the amount normally found in serum after zinc intoxication, symptoms range from none to severe anemia, growth failure, and systemic inflammation, and resemble zinc deficiency rather than chronic or acute intoxication [172–175]. The elevated zinc levels have been attributed to excessive binding to serum proteins, e.g., by albumin [171,173], or to overexpression of the zinc-binding S100 protein calprotectin [172,174]. Hence, the large amounts of zinc in the serum of these patients are sequestered by proteins, potentially even depleting biologically available zinc [175].

Clinical manifestations of moderate zinc deficiency are mainly found in patients with low dietary zinc intake, alcohol abuse, malabsorption, chronic renal disease, and chronic debilitation. Symptoms include growth retardation (in growing children and adolescents), hypogonadism in men, skin changes, poor appetite, mental lethargy, delayed wound healing, taste abnormalities, abnormal dark adaptation, and anergy [165].

Moderate zinc deficiency can also occur as a consequence of sickle cell disease [176]. Hyperzincuria and a high protein turnover due to increased hemolysis lead to moderate zinc deficiency in these patients, which causes clinical manifestations typical for zinc deficiency, such as growth retardation, hypogonadism in males, hyperammonemia, abnormal dark adaptation, and cell-mediated immune disorder [177] connected with thymic atrophy [178].

In mild cases of zinc deficiency, slight weight loss, oligospermia and hyperammonemia were observed [165]. One population in which mild zinc deficiency occurs with high prevalence, even in industrialized countries, are the elderly. Here, a significant proportion has reduced serum zinc levels, and zinc supplementation studies indicate that this deficiency contributes significantly to increased susceptibility to infectious diseases [44].

The overall frequency of zinc deficiency worldwide is expected to be higher than 20% [179]. In developing countries, it may affect more than 2 billion people [166,180–182]. Furthermore, it has been estimated that only 42.5% of the elderly (≥71 years) in the Unites States have adequate zinc intake [183]. This widespread occurrence combined with the variety of clinical manifestations makes zinc deficiency a serious nutritional problem, which has a far greater impact on human health than the relatively infrequent intoxication with zinc.

## 6. Conclusions

Zinc is an essential trace element, and the human body has efficient mechanisms, both on systemic and cellular levels, to maintain homeostasis over a broad exposure range. Consequently, zinc has a rather low toxicity, and a severe impact on human health by intoxication with zinc is a relatively rare event.

Nevertheless, on the cellular level zinc impacts survival and may be a crucial regulator of apoptosis as well as neuronal death following brain injury. Although these effects seem to be unresponsive to nutritional supplementation with zinc, future research may allow influencing these processes via substances that alter zinc homeostasis, instead of directly giving zinc.

Whereas there are only anecdotal reports of severe zinc intoxication, zinc deficiency is a condition with broad occurrence and potentially profound impact. Here, the application of “negative zinc”, *i.e.*, substances or conditions that deplete the body of zinc, constitute a major health risk. The impact ranges from mild zinc deficiency, which can aggravate infections by impairing the immune defense, up to severe cases, in which the symptoms are obvious and cause reduced life expectancy.
